# Motif Enrichment Analysis: a unified framework and an evaluation on ChIP data

**DOI:** 10.1186/1471-2105-11-165

**Published:** 2010-04-01

**Authors:** Robert C McLeay, Timothy L Bailey

**Affiliations:** 1 Institute for Molecular Bioscience, The University of Queensland, Brisbane, Queensland 4072, Australia

## Abstract

**Background:**

A major goal of molecular biology is determining the mechanisms that control the transcription of genes. Motif Enrichment Analysis (MEA) seeks to determine which DNA-binding transcription factors control the transcription of a set of genes by detecting enrichment of known binding motifs in the genes' regulatory regions. Typically, the biologist specifies a set of genes believed to be co-regulated and a library of known DNA-binding models for transcription factors, and MEA determines which (if any) of the factors may be direct regulators of the genes. Since the number of factors with known DNA-binding models is rapidly increasing as a result of high-throughput technologies, MEA is becoming increasingly useful. In this paper, we explore ways to make MEA applicable in more settings, and evaluate the efficacy of a number of MEA approaches.

**Results:**

We first define a mathematical framework for Motif Enrichment Analysis that relaxes the requirement that the biologist input a selected *set *of genes. Instead, the input consists of *all *regulatory regions, each labeled with the level of a biological signal. We then define and implement a number of motif enrichment analysis methods. Some of these methods require a user-specified signal threshold, some identify an optimum threshold in a data-driven way and two of our methods are threshold-free. We evaluate these methods, along with two existing methods (Clover and PASTAA), using yeast ChIP-chip data. Our novel threshold-free method based on linear regression performs best in our evaluation, followed by the data-driven PASTAA algorithm. The Clover algorithm performs as well as PASTAA if the user-specified threshold is chosen optimally. Data-driven methods based on three statistical tests–Fisher Exact Test, rank-sum test, and multi-hypergeometric test—perform poorly, even when the threshold is chosen optimally. These methods (and Clover) perform even worse when unrestricted data-driven threshold determination is used.

**Conclusions:**

Our novel, threshold-free linear regression method works well on ChIP-chip data. Methods using data-driven threshold determination can perform poorly unless the range of thresholds is limited *a priori*. The limits implemented in PASTAA, however, appear to be well-chosen. Our novel algorithms—AME (Analysis of Motif Enrichment)—are available at http://bioinformatics.org.au/ame/.

## Background

Elucidating the mechanisms that control the transcription of genes is one of the major goals of molecular biology. One current approach is to determine whether the regulatory sequences (e.g., promoters) of a set of genes have significantly higher than expected affinity for a regulatory protein or microRNA for which the DNA-binding motif is known. Such a motif is said to be "enriched" in the set of sequences. The regulatory proteins and microRNAs whose motifs are enriched in the set of regulatory sequences are candidate transcriptional regulators for some or all of the genes. The same approach can also detect motifs that predict significantly lower than expected DNA binding by a protein or microRNA to a set of regulatory sequences, indicating that binding may be detrimental to the proper regulation of some or all of the genes [1,2].

Looking for over- and under-represented known motifs in sets of genes is often referred to as "motif enrichment analysis" (MEA), and this name has been applied to a large variety of specific analysis methods. Generally, the set of genes has been identified using measurements of gene expression (e.g., expression microarray data) or measurements of transcription factor (TF) binding (e.g., chromatin immunoprecipitation on microarray (ChIP-chip) data). The known motifs have come from compendia of TF and microarray binding motifs such as JASPAR and Transfac. More recently, the advent of protein-binding microarray (PBM) data has lead to a rapid expansion in the coverage of TF binding motif databases [3]. The increasing number of known regulatory motifs, and the increasing availability and quality of expression data, means that the value of MEA as a tool for understanding transcriptional regulation is growing.

The above definition of MEA specifies that one of the inputs is a *set *of genes. This definition does not encompass methods where the input genes are labeled with some "signal" (e.g., microarray fluorescence signal), and the method considers gene sets of different sizes by thresholding the genes on the given signal. Since the scientist using MEA would normally identify a set of genes in just this way—e.g., by including in the input set all genes with expression or binding signal greater than some threshold—such "partitioning" methods are an attractive generalisation of our initial definition of MEA. They are strict generalisations of our previous definition as long as the scientist can restrict the range of thresholds the method considers. They are attractive because they remove the burden of choosing the optimum gene set threshold from the scientist, potentially resulting in more significant associations being detected.

Because of the increasing value of MEA as a scientific tool, we undertake a comparative study of the accuracy of four major approaches. To unify our study, we present a formulation of MEA that encompasses methods that automatically determine the optimal gene set given a larger set of genes, perhaps all genes in the organism of interest, each labeled with some kind of biological signal, typically a microarray intensity signal. The MEA methods we study include examples of the most popular types in extensive use, including the popular Fisher Exact Test [4]; its extension, the multi-hypergeometric test [5]; the rank-sum test [6,7]; and Clover [1]. We also study two threshold-free methods—linear regression and Spearman's rank correlation. Linear regression is an approach that has seen little or no application in MEA, although it has been used extensively in the related task of *ab initio *motif discovery [8,9]. To our knowledge, Spearman's rank correlation has not been previously used for either MEA or motif discovery. For completeness, we study both fixed-threshold versions and partitioning versions that optimize over the biological signal threshold of each of these MEA methods, with the exception of the linear regression and Spearman's rank correlation methods, which use no threshold.

In order to evaluate the accuracy of MEA methods, we use transcription factor binding data from yeast ChIP-chip experiments. We use only experiments where the DNA-binding motif for the transcription factor is known, and measure how the MEA method ranks the known motif of the ChIP-ed TF. This provides us with an objective measurement of the quality of the predictions made by different MEA approaches. Although we compare MEA approaches using microarray-based TF binding data, we emphasize that the results are likely to extend to other applications such as microarray-based gene expression data. This conclusion is supported by the fact that the results of MEA analysis of yeast ChIP-chip data were shown previously to agree with MEA analysis of yeast expression data [10].

The results of our study also shed light on discriminative approaches to *ab initio *motif discovery. Such motif discovery methods differ from MEA mainly in that they do not restrict their search to a compendium of known motifs. Rather, they typically search an extremely large space of possible motifs, looking for the motif that maximizes an "objective function". The Fisher Exact Test, the multi-hypergeometric test and linear regression have all been employed as objective functions in motif discovery algorithms. (To our knowledge, there is no motif discovery method similar to the Clover MEA method.) Our results are relevant to motif discovery because any objective function that cannot distinguish the correct DNA-binding motif from among a small set of motifs in the MEA task will clearly do poorly in the motif discovery setting.

### A Mathematical Framework for Motif Enrichment Analysis

In order to frame our comparative study and make it easier to see the relationship among existing MEA approaches, we present a generalised formulation of the MEA task. Our definition of MEA is expansive—any method that attempts to measure the association (positive or negative) between an *in silico *"motif score" of a DNA sequence and a biological "signal" associated with that sequence. In a typical application of MEA, the motif score might be 1 if the sequence contains a match to a particular *k*-mer (the motif) and 0 otherwise, and the biological signal might be the fluorescence intensity of the DNA probe containing the sequence on a microarray. In general, a motif can be represented as a position-weight matrix (PWM), which can express any score function for *k*-mers, (fixed-length) regular expressions and more powerful energy-based scoring functions [11].

To define a particular MEA method in our framework, we specify three things: a motif affinity score function, *f*, an association function, *F*, and an association score, *R*. The input to the method is a set of DNA sequences, each of which is associated with a (numerical) biological signal intensity, *Y*. The motif affinity score function, *f*, is used to calculate the score, *X*, of each DNA sequence in the input. The association function, *F*, is then used to compute the degree of association between *X *and *Y *over the entire input set. Without loss of generality, we assume that larger values of *F *indicate a stronger positive association between *X *and *Y*. In general, the association function will require partitioning the input set by thresholding on either or both *X *and *Y*, so the final score in our framework is the association score, *R*, which computes the maximum of *F *over a user-specified range of partitioning thresholds. In some methods, the affinity function will also require a threshold, and *R *will maximise *F *over that threshold as well as over those for *X *and *Y*. For clarity, we omit the obvious generalisation of *R *for negative associations, which requires minimizing *F*. Let(1)

be the input sequences and their associated biological signals. We map the sequences to their affinity for the motif using the affinity function *f*, where(2)

The parameter *t*_*m *_may be unused by the function, but it is typically a threshold used if the value of the function is discrete (e.g., the number of "matches" to the motif in the sequence). Alternatively, the value of *f *may be continuous (e.g., the total binding affinity of a TF for the given sequence). We then define a "mapped" dataset in terms of the affinity and the signal as(3)

The degree of association between motif *M *and signal *Y *is computed using the "association function" *F*,(4)

The parameters *t*_*x *_and *t*_*y *_are thresholds used by some forms of the association function to partition the data points according to their *X *and *Y *values, respectively. Finally, the "association score" for our definition of MEA is(5)

where *r*_*m*_, *r*_*x*_, and *r*_*y *_are the legal *ranges *(or sets of values) for *t*_*m*_, *t*_*x *_and *t*_*y*_, respectively. Some or all of these three parameters may be ignored by a particular affinity score function, *R*. In particular, partition-free methods ignore all three partition threshold parameters.

## Methods

We evaluate six basic MEA approaches and, where possible, variants of the approaches that optimize the association function over different ranges of the biological signal threshold, *t*_*y*_. Most of the association functions do not allow for maximising over *X*, so we do not study maximisation over that dimension. We also do not examine MEA approaches that maximise over *t*_*m *_in this work. Adapting the methods selected for this study to maximise over *t*_*m *_would considerably increase the run-time of these methods. In this section, we describe the motif affinity functions, association functions and association scores that we use. We then detail the specific MEA approaches whose accuracy we measure. Finally, we describe our evaluation methodology in detail.

### Motif affinity functions

The motif affinity function, *f*(*S*_*g*_, *M*, *t*_*m*_) (Eqn. 2), is used in MEA to assign a motif affinity score, *X*_*g*_, to a DNA sequence, *S*_*g*_. The score represents the affinity for the sequence of a DNA-binding molecule with binding motif *M*. The most commonly used motif affinity functions either count the number of "matches" to a motif in the DNA sequence or compute some function that represents the total binding of the TF or microRNA to the sequence. We study both of these types of affinity function and, in both cases, we represent the motif, *M*, by a log likelihood ratio PWM [11]. All motif PWMs were generated using a uniform background model in the denominator of the likelihood ratio.

When counting matches, we use FIMO [7], which scores each position in a sequence, *S*_*g*_, (on both strands) using the PWM, *M*, and computes the *p*-value of each score. (The *p*-value is based on a zero-order Markov model of the input sequences.) The value of the affinity function, *f*(*S*_*g*_, *M*, *t*_*m*_), is the number of positions the sequence with *p*-value less than or equal to *t*_*m*_, the motif score threshold. We refer to this motif affinity function as "MC" (for "match-count").

For our other motif affinity function, which estimates the total binding of the TF or microRNA represented by the motif, we use the AMA algorithm [12] to compute the average motif affinity (AMA) score of the sequence, *S*_*g*_, to the motif, *M *[13]. The AMA score is equal to the average *likelihood ratio *(not the log likelihood ratio) of the sequence (on both strands). We use a minor variant of the AMA score, which we call RMA (for relative motif affinity), when computing the linear regression association function (see below). To compute RMA, we divide the AMA score by the maximum possible AMA score of a single position in any sequence. This ensures that the range of the binding affinity function is [0,...,1]. No motif match threshold (*t*_*m*_) is required when using AMA as the motif affinity function.

### Association functions

The association function, *F*(, *t*_*x*_, *t*_*y*_) (Eqn. 4), computes the degree of association between the motif affinity score, *X*, and the biological signal, *Y*, which in some cases requires partitioning the (mapped) points, , in the *X *and/or *Y *dimensions using thresholds *t*_*x *_and *t*_*y*_, respectively. We design our association functions so that a larger value implies a stronger positive correlation between *X *and *Y*. For our first three association functions, the value of the function is the reciprocal of the *p*-value of a statistical test. The last two association functions compute non-statistical scores.

The first association function we study—the Fisher Exact Test—is perhaps the most frequently used association function in MEA approaches. When using the Fisher Exact Test, we create the 2-by-2 contingency table induced on the points by the thresholds *t*_*x *_and *t*_*y*_, and compute the *p*-value of the observed (or greater) number of points where *X*_*g *_≥ *t*_*x *_and *Y*_*g *_≥ *t*_*y*_. This is done using the hypergeometric distribution density function [14]. As noted above, we use the reciprocal of the *p*-value as the value of the association function.

Our second association function–the multi-Hypergeometric (mHG) Test—extends the Fisher Exact Test to multiple dimensions [15]. It requires that the affinity function have integral values in some fixed range [0,..., *c*]. We split the points in the *Y *dimension using the *t*_*y *_threshold, and compute the *p*-value of the observed distribution (or more extreme) of *X*_*g *_values in the points with *Y*_*g *_≥ *t*_*y *_using the multi-hypergeometric distribution. The value of *t*_*x *_is ignored by this association function.

Our third association function—the rank-sum test—also ignores the value of *t*_*x*_. Instead, we sort the mapped points on *X*, and compute the sum of the ranks of points where *Y*_*g *_≥ *t*_*y*_. We then compute the *p*-value that the sum of the ranks is as small or smaller than the observed value in the standard manner [16].

The fourth association function we study is the score computed by Clover (see [1], Eqn. 4). This association function can only be computed using the AMA motif affinity function, and its value is essentially the average of the motif affinity function over all possible subsets of points where *Y*_*g *_≥ *t*_*y*_. As with the previous two association functions, the value of *t*_*x *_is ignored by this function.

Our fifth association function is based on the mean-squared error of the linear, least-squares fit to the mapped data points. We assume a linear relationship between *X *and *Y*,(6)

and perform least-squares regression on all mapped points. The value of the linear regression association function (LR) is(7)

where *E *is the mean-squared error of the fit to the mapped data points, and sgn(*m*) is a function that returns -1 if *m *is less than 0, and 1 otherwise. (The dots in the function definition indicate that those arguments are not used.) This definition of *F *insures that its value is large and positive when there is a strong, positive correlation between *X *and *Y*. Note that to measure a negative association between *X *and *Y*, we could use -sgn(*m*) times the reciprocal of the mean-squared error in Eqn. 7. In the current study, however, we are only interested in positive correlations between the motif affinity score, *X*, and the biological signal, *Y*.

The final association function that we study is Spearman's rank correlation [17]. Like the linear regression association function, Spearman's rank correlation is threshold-free on both *X *and *Y*. Unlike linear regression, however, Spearman's rank correlation coefficient is a non-parametric measure of correlation. It does not assume a linear relationship between *X *and *Y*, rather, it assesses the degree to which an arbitrary monotonic function can describe the relationship between *X *and *Y*.

### Specific MEA methods evaluated

Each of the MEA methods that we evaluate consists of plugging one of our six association functions, *F *(Eqn. 4) into the association score function, *R*(*D*, *M*, *r*_*m*_, *r*_*x*_, *r*_*y*_) (Eqn. 5). To fully specify an MEA method, we must also specify which motif affinity function we use, and the ranges (*r*_*m*_, *r*_*x *_and *r*_*y*_) of motif threshold, affinity function threshold and biological signal threshold over which we maximise *F*.

We designate each of our MEA methods using a name indicating the type of association function it uses. In this work, we only study maximizing over the *Y *threshold, *t*_*y*_, where the range, *r*_*y*_, either consists of a single value—"*Y*-fixed-partition" (YFP); all values greater than a given value—"*Y*-constrained-partition-maximisation" (YCPM); or all possible values—"*Y*-unconstrained-partition-maximisation" (YUPM). In what follows, we designate each of our MEA methods using a name indicating the type of association function and type of partition maximisation it uses, e.g., "Fisher-YFP". The specific methods we test are summarized in Table 1. Also included in the table is the MEA algorithm PASTAA [18], which we include in our evaluation for completeness. Unlike the other methods tested here, PASTAA [18] performs constrained partition maximisation jointly over both motif affinity function scores (XCPM) and biological signal (YCPM), maximising its association function–the Fisher Exact Test.

**Table 1:** Specific MEA methods tested in this study.

*Method Name*	*Motif Affinity Function (X_g_)*	*Biological Signal (Y_g_)*	*Partition Maximization Variants*
Fisher	MC: {0, 1}	1/*p*	YFP, YCPM, YUPM
mHG	MC: {0, 1, 2}	1/*p*	YFP, YCPM, YUPM
Ranksum	AMA	1/*p*	YFP, YCPM, YUPM
Clover	AMA	1/*p*	YFP, YCPM, YUPM
PASTAA	AMA-like	1/*p*	X, YCPM
Spearman	AMA	1/*p*	none
LR	RMA	-log(*p*)	none

The method names refer to the association function they use: "Fisher" (Fisher Exact Test), "mHG" (multi-hypergeometric test), "Ranksum" (Ranksum or Mann-Whitney U Test), "Clover", "Spearman" (Spearman's rank correlation coefficient) and "LR" (linear regression). For Fisher, the MC motif affinity function is capped at 1 match; for mHG, the MC function is capped at 2 matches. We set *t*_*m *_= 0.0002 for the MC motif affinity function (i.e., matches have PWM score with *p*-value less than 0.0002). In all methods except LR, *Y*_*g *_is defined as 1/*p*, where *p *is the *p*-value of a microarray probe fluorescence signal; for the LR method, *Y*_*g *_is defined as *p*.

### Evaluation of MEA methods

To compare the different MEA methods, we study their ability to correctly identify the known motif for a yeast TF from microarray fluorescence data from a ChIP-chip experiment involving that TF. We utilize the data from a large set of ChIP-chip experiments for which the DNA binding motif of the ChIP-ed TF is known. Our evaluation data consists of these ChIP-chip datasets, and the set of known motif PWMs, *L*, for the TFs used in the experiments. As noted in Table 1, we use the reciprocal of the *p*-value of the fluorescence score of a microarray probe as the biological signal, *Y*_*g*_. The DNA sequence of the probe is used as *S*_*g *_in computing the motif affinity, *X*_*g*_.

We test an MEA method on a given ChIP-chip dataset, *D*, as follows. We use the MEA method to compute the the association score *R*(*D*, *M*, *r*_*m*_, *r*_*x*_, *r*_*y*_) (Eqn. 5) for for each of the known TF motifs, *M *∈ *L*. Ideally, the motif of the ChIP-ed TF, *M*_*k*_, will have the highest association score among all the TF motifs, so we measure accuracy using a metric based on the *rank *of the ChIP-ed TF's motif's score among the scores for all motifs. We call this metric "percentile rank accuracy" (PRA), and it ranges from 0 to 100, with 100 being ideal–the ChIP-ed TF's motif has the highest association score. When the association scores for each of the *N *known motifs are sorted in *increasing *order (so that the most positively correlated motif has rank *N*), the PRA accuracy measure is defined as(8)

where *R*_*k *_is the rank of the ChIP-ed TF's motif, *M*_*k*_.

Our set of datasets consists of the 237 yeast TF ChIP-chip experiments performed by Harbison *et al*. [19] for which the DNA binding motif of the ChIP-ed TF was reported by MacIsaac *et al*. [20]. Our set of known motifs, *L*, consists of these 124 TF PWM motifs. Each ChIP-chip dataset contains the probe sequences, *S*_*g*_, and their fluorescence *p*-values, *Y*_*g*_, where the microarray probes consisted of all yeast (*Saccharomyces cerevisiae*) intergenic regions. As noted above, previous MEA studies of these datasets have yielded results in agreement with MEA performed on yeast expression microarray datasets [10]. Unlike previous studies [18], which selected a subset of motifs to test, we test the complete set of ChIP-chip data for which a known motif exists. When using the linear regression (LR) MEA method, we do not remove outliers or check for skewness, unlike the use of linear regression for *ab initio *motif discovery in Foat *et al*. [9]. They also used the fluorescence ratio as the biological signal, whereas we use the inverse of the logarithm of the fluorescence *p*-value.

We create likelihood ratio motifs from the known motif *sites *provided by MacIsaac *et al*. [20] in the standard manner [11] using the "tamo2meme" script provided with the MEME Suite [7]. We use a "pseudocount" of 0.25 when estimating the numerator of the likelihood ratio from the counts of each DNA base in each position in the motif in the known sites, and use a zero-order background model estimated from all *Saccharomyces cerevisiae *intergenic regions using the "fasta-get-markov" script included in the MEME Suite.

## Results

### Fixed-partition methods

**Figure 1:**
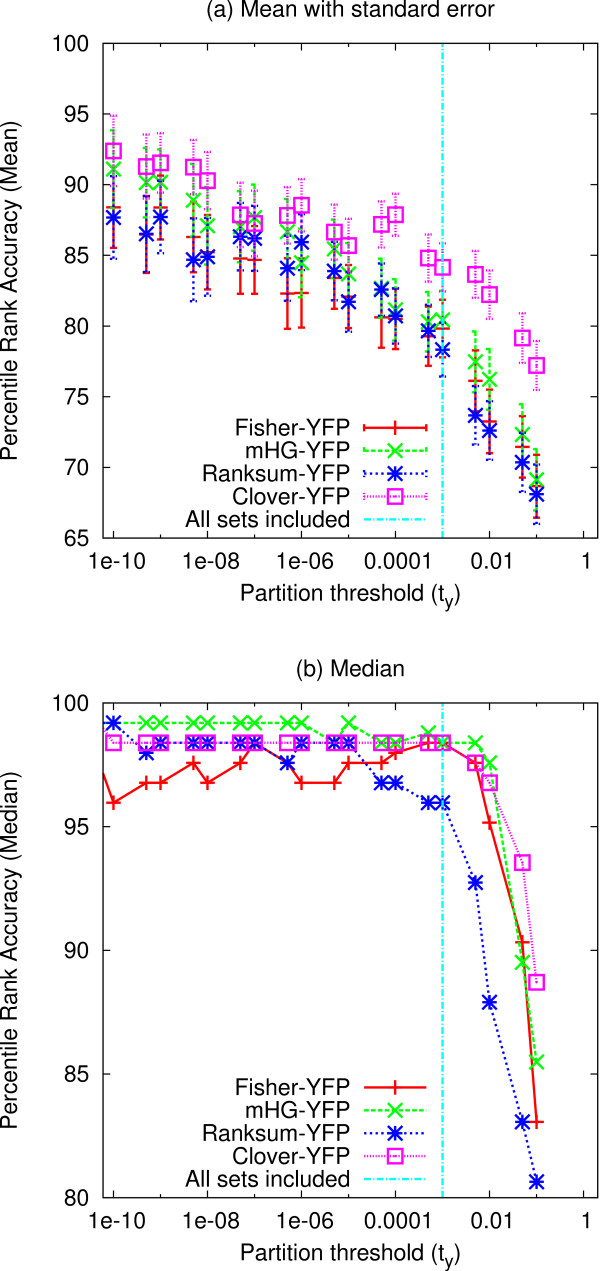
**Accuracy of MEA methods using fixed *Y *partitions**. The ability of different MEA methods to correctly rank the known TF motif in 237 yeast ChIP-chip experiments is shown. Each point corresponds to the mean (Panel **a**) or the median (Panel **b**) percentile rank accuracy (PRA) of an MEA method on all ChIP-chip datasets that contain at least one sequence with a fluorescence *p*-value less than the value of *t*_*y *_(*X*-axis). Increasing *X *values correspond to relaxing the threshold for a sequence to be considered bound by a TF. To the right of the vertical line, all 237 sets are included; to the left, increasingly fewer sets are included at stricter *t*_*y *_thresholds.

We first explore the accuracy of the simplest MEA methods we consider in this study, the YFP methods. These methods split the input data into positive and negative sets using a fixed threshold on the biological signal, *Y*. Fixed-partition MEA methods have been extensively used (e.g., [21]). We measure the accuracy of four YFP methods—Fisher-YFP, mHG-YFP, Ranksum-YFP and Clover-YFP—on the task of identifying the correct TF motif in each of the 237 yeast ChIP-chip datasets. The biological signal, *Y*, is the ChIP-chip fluorescence *p*-value, and we run each MEA method using various values of the fixed *Y *partitioning threshold, *t*_*y*_. The results of this experiment are shown in Fig. 1. Note that the results to the left of the vertical blue line in the figure are for increasingly smaller subsets of the 237 ChIP-chip datasets since we ignore all datasets where the partition threshold on *Y*, *t*_*y*_, results in an empty positive set. For example, the points in the figures with *t*_*y *_= 10^-10 ^give results for the 57 ChIP-chip datasets containing at least one fluorescence *p*-value less than 10^-10^.

The YFP version of Clover is clearly superior to the other methods at identifying the ChIP-ed TF motif in all 237 yeast ChIP-chip datasets (Fig. 1a). The mean accuracy (PRA, Eqn. 8) of all the methods increases with decreasing *Y *partition threshold. At a threshold of *t*_*y *_= 0.001, the smallest partition threshold that can be used with all 237 datasets, Clover-YFP ranks the correct TF in the 84^th ^percentile (PRA = 84.1), while the next best method (mHG-YFP) ranks it in the 80^th ^percentile (PRA = 80.4), on average. The superiority of Clover-YFP is even more pronounced at larger values of the *Y *partition threshold, but the absolute accuracy of all methods decreases as the partition threshold increases (Fig. 1a).

The YFP version of the Ranksum method is clearly the worst of the methods tested on all 237 ChIP-chip datasets. Even under the more forgiving median PRA metric, which places less emphasis on datasets where a method performs extremely poorly, Ranksum-YFP has substantially lower accuracy than the other methods (Fig. 1b). At a *Y *partition threshold of *t*_*y *_= 0.001, the median PRA for Ranksum-YFP is only 96.0, while it is 98.4 for the three other MEA methods tested. Since both Clover-YFP and Ranksum-YFP use AMA as the motif *affinity *function (Table 1), Clover's *association *function is clearly better than the rank-sum test for MEA using a fixed *Y *partition, at least on this type of biological signal data (ChIP-chip). None of the YFP versions of the MEA methods we test here perform extremely well on all 237 yeast ChIP-chip datasets. In fact, no method places the ChIP-ed TF motif among the top three predicted motifs for more than 60% of the ChIP-chip datasets (data not shown). This is not surprising, given that Gordân *et al*. [22] found that in 35% of the ChIP-chip experiments no PBM-derived (an independent, *in vitro *method of determining motif sequence specificity) was significantly enriched.

The Clover-YFP method is also more accurate than the *Y *partition maximization variants of the other three MEA methods when tested just on the yeast ChIP-chip datasets containing fluorescence *p*-values below *t*_*y *_= 0.001 (results to left of vertical blue line in Fig. 1a). However, the relative difference among the methods in terms of mean PRA decreases with decreasing *Y *partition threshold. Thus, among YFP variants of the MEA methods, Clover-YFP appears to be the best approach for ChIP-chip data, and is especially advantageous when the ChIP-chip data has low-signal-to-noise ratio (i.e., in cases where no microarray probe has a low fluorescence *p*-value).

### Unconstrained partition maximization methods

We see from our fixed-partition experiments that the accuracy of MEA methods on the yeast ChIP-chip TF identification task depends strongly on the choice of fluorescence *p*-value threshold. In those experiments we follow common practice and choose a single threshold for all 237 ChIP-chip datasets. We wondered if it would be advantageous to choose a different, data-dependent threshold for each dataset. One way to do this automatically is to consider all possible thresholds and choose the one that maximizes the association function (Eqn. 4). This *type *of approach has been investigated recently for the Fisher [18] and mHG [15] association functions for MEA and motif discovery, respectively. (As we discuss later, those two studies used forms of *constrained *rather than unconstrained partition maximization.)

**Figure 2:**
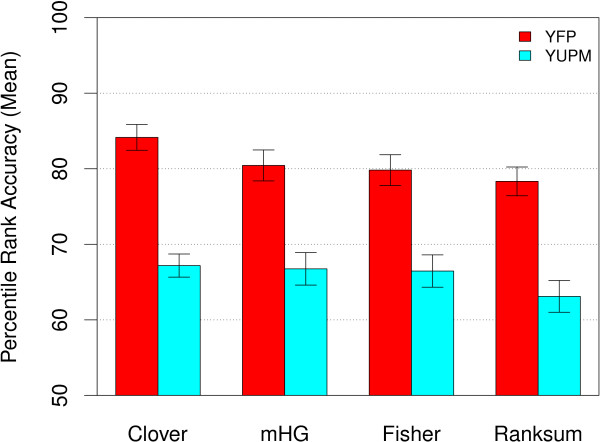
**Accuracy of MEA methods using unconstrained-*Y*-partition-maximisation**. The ability of different MEA methods to correctly rank the known TF motif in 237 yeast ChIP-chip experiments is shown. The mean percentile rank accuracy of unconstrained-*Y*-partition-maximization (YUPM, blue bars) and fixed-partition (YFP, red bars, *t*_*y *_= 0.001) variants of four MEA methods is shown. Error bars show standard error.

Unconstrained *Y *partition maximization (YUPM) fails to improve all four MEA methods' abilities to identify the ChIP-ed yeast TFs in the 237 ChIP-chip datasets (Fig. 2). Compared with using the smallest fixed *Y *threshold such that all 237 ChIP-chip datasets have at least one positive sequence (*t*_*y *_= 0.001), allowing the methods to choose the partition threshold according to Eqn. 5 results in substantially lower average accuracy (mean PRA). For example, the YUPM version of Clover (Clover-YUPM) has mean PRA of 67.19, compared with 84.15 when we fix the *Y *threshold at 0.001 (Clover-YFP). This is in fact the best mean accuracy of any of the YUPM methods on the 237 yeast ChIP-chip datasets. Interestingly, the Ranksum MEA method, in addition to being the poorest method when using YFP, decreases the most in accuracy when YUPM is used.

The YUPM variants of the MEA methods consider every possible partitioning of the data sorted according to the biological signal, *Y*. At least for ChIP-chip data, it is clear from Fig. 2 that choosing the *Y *partition that maximizes the association function is *not *a good idea. Inspection of the data underlying Fig. 2 shows that highly-ranked motifs (other than the correct motif) often have maximal association scores for *Y *partitions with extremely large numbers–much larger than the TF would be *a priori *expected to bind–of "positive" sequences (data not shown). Most of these "positive" sequences have very large *Y *values and the large association score is due to a slight correlation between *X *(the motif affinity score) and *Y *(the ChIP-chip fluorescence *p*-value) over many sequences. The association functions are quite good at detecting such correlations, but the correlations are often not indicative of functional binding of the TF, as indicated by the lower accuracy of the YUPM variants of MEA in Fig. 2.

### Constrained partition maximization methods

As mentioned above, the unconstrained partition maximization MEA methods seem to perform poorly on the yeast TF identification task due to choosing optimal *Y *(ChIP-chip fluorescence *p*-value) thresholds corresponding to very large "positive" sets of sequences. This may explain why previous uses of partition maximization for MEA and motif discovery have often constrained the maximum size of the positive set. For example, the MEA algorithm PASTAA [18] limits the size of the positive set to no more than 1000 sequences. Similarly, the motif discovery algorithm DRIM [15], which was tested on the yeast ChIP-chip data used in the current study, limits the positive set to at most 300 sequences by default, and no more than 1000 sequences. These are both only small fractions of the total number of sequences (about 6000) in the yeast ChIP-chip datasets used here.

**Figure 3:**
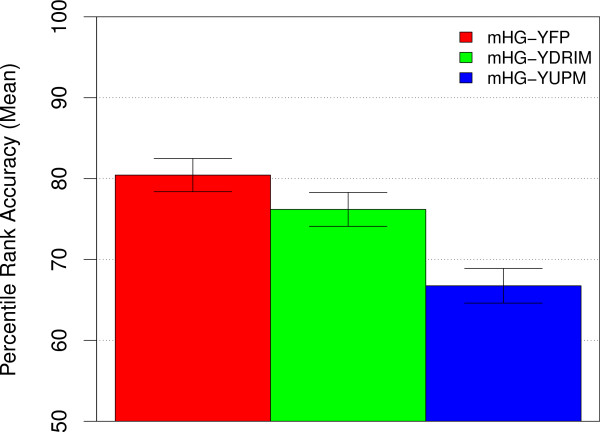
**Accuracy of the mHG method constrained to at most 300 positive sequences**. The ability of three variants of the mHG method to correctly rank the known TF motif in 237 yeast ChIP-chip experiments is shown. Each bar represents the mean PRA of versions of an MEA method. The bar labeled mHG-YDRIM shows accuracy using partition maximization, limited to partitions with a maximum of 300 "positive" sequences. The other two bars show accuracy using the fixed partition method with *t*_*y *_= 0.001 (mHG-YFP) and and unconstrained partition maximisation (mHG-YUPM), respectively.

If we constrain our partition maximization variant of the mHG method to *Y *thresholds yielding no more than 300 "positive" sequences, the mean accuracy on the yeast TF ranking task is intermediate between the fixed partition and unconstrained partition maximization variants (Fig. 3). Thus, on this task, the type of constrained partition maximization used by DRIM does not seem to improve on using a fixed partition corresponding to assigning sequences with fluorescence *p*-values less than 0.001 to the "positive" set. We note that in the 237 yeast ChIP-chip datasets, the mean value of *Y *for the 300^th ^sequence (sorted by increasing *Y*, fluorescence *p*-value) is 0.04. This means that limiting the *Y *partition to 300 "positive" sequences allows sequences with less significant biological signals (*Y*) to be included in the "positive" set, compared with the fixed threshold of *t*_*y *_= 0.001 we use with method mHG-YFP in Fig. 3.

Perhaps a more general way to constrain the partition maximization methods is to state the constraint in terms of the biological signal *Y*, rather than as a number of "positive" sequences. This approach is described by Eqn. 5, where we place an *upper bound *on the *Y *threshold, *t*_*y*_, but no lower bound. (That is, we define *r*_*y *_= [0, *b*] for some upper bound, *b*, in Eqn. 5.) This effectively limits the maximum size of the "positive" sequence set, but in a data-dependent manner. In the current application, all sequences with ChIP-chip fluorescence *p*-values less than *t*_*y *_*may *be included in the "positive" set, but none with larger *p*-values.

**Figure 4:**
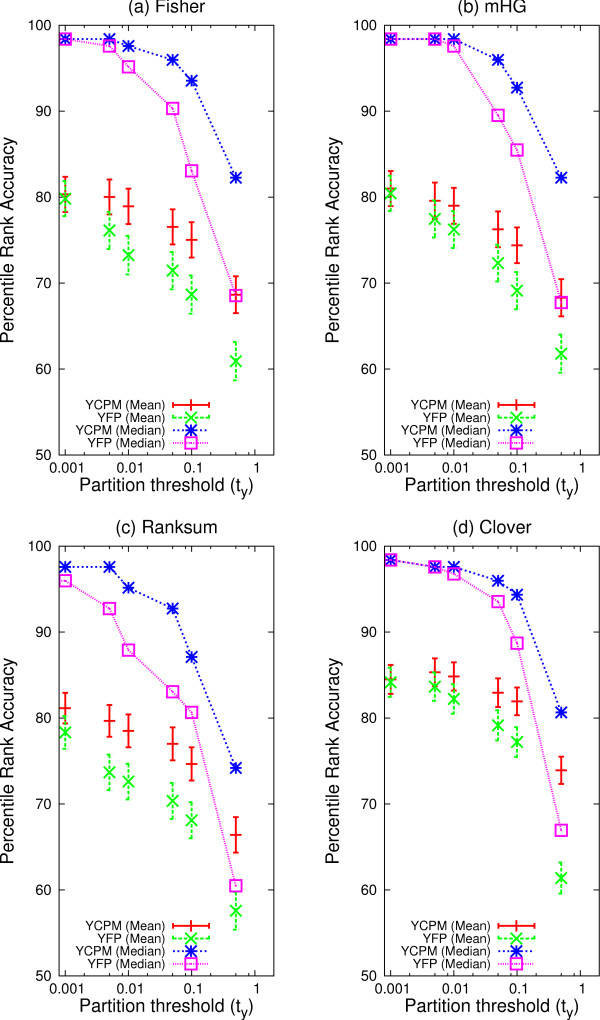
**Accuracy of MEA methods using constrained partition-maximization**. The ability of different MEA methods to correctly rank the known TF motif in 237 yeast ChIP-chip experiments is shown. Each panel shows the accuracy of the *Y *constrained partition maximization (YCPM) of a method, along with the fixed partition (YFP) variant's accuracy for comparison. Each point shows the mean or median PRA (*Y*-axis) of the MEA method. For YCPM methods, the *X*-axis of the plot is the *maximum *value, *b*, that *t*_*y *_may assume; for YFP methods, it is the method's fixed threshold, *t*_*y*_.

The maximum accuracy of the constrained *Y *partition maximization variants of three out of four MEA methods is no better than that of the fixed partition variants on the yeast ChIP-chip TF motif identification task (Fig. 4). There is a slight improvement in the worst method (Ranksum) when the upper bound on *t*_*y *_is set to 0.001, but it remains the least accurate method on this task. As we increase the value of *b *(and, hence the maximum size of the "positive" set), both the mean and median percentile rank accuracy of all four YCPM methods fall. The best accuracy for the constrained methods is achieved when the upper bound on *t*_*y *_is 0.001, the smallest possible bound in order that all 237 ChIP-chip datasets have at least one "positive" sequence.

However, the constrained *Y *partition maximization (YCPM) MEA variants are more *robust *than the fixed partition (YFP) variants. Both variants have one free parameter that must be chosen by the user–the upper bound, *b *for the YCPM variants, and the fixed threshold, *t*_*y*_, for the YFP variants. It is clear from Fig. 4 that the YCPM variants are less sensitive to the relaxation of the maximum selectable threshold to consider a TF bound to a sequence (*b*) than the YFP variants are to the relaxation of the absolute threshold to consider a TF bound to a sequence, *t*_*y*_. Since the user will not generally know the optimum choice for the free parameter for either method, this is a clear advantage for the constrained *Y *partition maximization variants of the four MEA methods compared with the fixed partition versions. What is more, Fig. 4 shows that the YCPM variants always achieve equal or better accuracy for a given value of the free parameter (*b*) compared to the YFP variants using the same free parameter value (*t*_*y*_). Hence, on the task studied here, the constrained *Y *partition maximization MEA variants are clearly superior to the fixed partition variants.

### Partition-free MEA methods

The advantage of the constrained partition maximization MEA variants (relative to the fixed partition variants) lies in their relative insensitivity to the choice of a single free parameter. However, a method of comparable accuracy with *no *free parameters that the user must choose would be better still. The unconstrained partition maximization variants have no free parameters, but perform very poorly on the current task, as we show above. One other parameter-free MEA method we study here is the linear regression (LR) method, which does not partition the sequences into "positive" and "negative" sets using the biological signal *Y*. Instead, the association function is the reciprocal of the error of the linear regression of *Y *and *X*.

**Figure 5:**
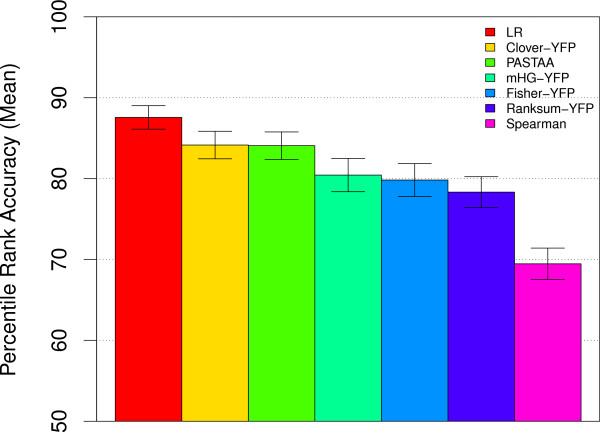
**Accuracy of a partition-free MEA method**. The ability of different MEA method to correctly rank the known TF motif in 237 yeast ChIP-chip experiments is shown. Each bar shows the mean PRA of the given MEA method on all 237 ChIP-chip datasets. Error bars show standard error. The LR method is partition free. PASTAA uses *X *and *Y *constrained partition maximization with a maximum of 1000 sequences in the "positive" sets. All fixed-partition (YFP) methods use a threshold of *t*_*y *_= 0.001.

Our *parameter-free *linear regression (LR) MEA method achieves higher accuracy on the yeast TF motif ranking task than each of the other four methods using the *optimal *values of their free parameters (Fig. 5). The LR method achieves a mean percentile rank accuracy of 87.57 compared with 84.15 for Clover-YFP, the second best method. It should be emphasized that this is an *unfair *comparison (to LR), since we have "cheated" for Clover-YFP, mHG-YFP and Ranksum-YFP by choosing the value of their free parameter (*t*_*y*_) that achieves the highest accuracy. It is likely that an actual user of one of these other methods (or the more robust YCPM variants) would not know the optimal parameter value, so their accuracy would be worse.

As the LR method performed strikingly well, we implemented another parameter-free method, Spearman's rank correlation coefficient. Unlike linear regression, Spearman's rank correlation does not suppose a linear relationship between *X *and *Y*. Our Spearman's rank correlation method performed extremely poorly, achieving a mean percentile rank accuracy of 69.46, the worst in this comparison, and substantially lower than the YFP methods.

Earlier, we mentioned that the MEA method PASTAA uses a form of constrained *Y *partition maximization. In fact, it performs constrained maximization over *both X *and *Y*, using an affinity function similar to AMA and the Fisher Exact Test association function. When applied to the TF ranking task, PASTAA (using its default constraints) performs better than all the other partition-based approaches except Clover (Fig. 5). This indicates the robustness of PASTAA, as we did not optimize its free parameters as we did in the case of the YFP variants of the other methods (including Clover). Nonetheless, on the yeast ChIP-chip TF motif ranking task, PASTAA achieves substantially lower accuracy compared to the partition- and parameter-free LR method we introduce here.

### Software Availability

We have released the two software tools developed in this study, and made them available online. AME (Analysis of Motif Enrichment) and RAMEN (Regression Analysis of Motif ENrichment) are both available for download from http://bioinformatics.org.au/ame/. Both AME and RAMEN are available as binaries for Mac OS X and Linux, with source available on request. Both tools are licensed under the MEME [7] license.

AME implements the Fisher, mHG, Ranksum, linear regression (LR), and spearman's rank correlation methods in YFP and YUPM modes. With an additional analysis step, AME can also be used for YCPM. RAMEN implements our parameter-free LR method and additionally supports the calculation of permutation-based *p*-values. More complete documentation for AME and RAMEN can be found on the website.

## Discussion

We have proposed a general definition of motif enrichment analysis (MEA) that encompasses a wide variety of existing approaches. In our definition, MEA consists of looking for an association of a sequence-based predicted binding score (the motif affinity function) and some other biological "signal" associated that is available for each sequence. Typically the motif affinity function would be based on a TF DNA-binding motif, but it could also be computed from a look-up table representation of the TF's affinity, such as those created by PBM experiments [23]. The biological signal will often be some measure of DNA-binding affinity as well (e.g., from ChIP-chip or ChIP-seq data), but it could also be a measure of an indirect affect of DNA-binding (e.g., mRNA expression level). Our definition of MEA also encompasses motif-based gene set analysis [12,21,24] since a gene set defines a function with values 0 or 1 on all genes. In other words, gene set analysis uses a biological signal that is '1' for genes in the set, and '0' for all other genes.

Our results suggest that the association function can have a large affect on the accuracy of motif enrichment analysis results. Somewhat surprisingly, the rank-sum test performed poorly compared to the Fisher Exact test, even though the latter test potentially ignores relevant data by considering only the number of genes above the signal threshold and not their ranks (see also [2]). The rather *ad hoc *(but clever) association function implemented in Clover performs as well or better than all other approaches except linear regression. Although we only study ChIP-chip data for yeast transcription factors here, we suspect that Clover may prove to be more robust than linear regression when MEA is applied to other types of biological signals such as mRNA expression where a linear relationship between DNA-binding and the biological signal is less likely to hold [9]. Nonetheless, for direct DNA-binding data such as ChIP, our novel linear regression MEA method (LR) appears extremely competitive with existing approaches such as Clover and PASTAA. Like PASTAA, our LR method has the additional advantage of not requiring the user to choose the threshold on the biological signal for partitioning the genes into positive and negative sets.

Several of the MEA association functions explored here require that the data be partitioned into sets. The appealing idea of maximizing the association function over all possible partitioning of the data according to the biological signal (*Y *unconstrained partition maximization, YUPM) proved to be a very bad approach. This was somewhat surprising, given that this approach has been reported as the basis for a motif discovery algorithm (DRIM [15]). In order to be successful, *Y *partition maximization must be constrained to only split the data according to a limited range of biological signal strengths [18]. This requirement essentially forces the user of MEA to somehow determine the best partition, obviating the advantage that would come from an algorithm that could determine the best threshold automatically from the input data. Nonetheless, the variant of constrained *X *and *Y *partition maximization employed by the PASTAA MEA algorithm works very well on the datasets tested here.

In the current work we have compared two existing MEA algorithms (Clover and PASTAA) and a range of variations of existing and novel methods. For example, OFTBS [25] is somewhat similar to Clover, and we tested the mHG test, used by PRIMA [5]. The rank-sum method that we test is similar to the rank-based measures used by ASAP [26], and to a method tested (and discarded) for use in PScan [2]. PAP [27] also uses a rank-based method.

Previous evaluations of MEA algorithms have often relied on anecdotal evidence to support the accuracy and benefits of the approach and method implemented. For example, Roider *et al*. [18] performs a very small scale analysis of PASTAA, comparing it to Clover [1], PAP [27] and a method similar to oPossum [28]. The methods are compared by scanning 25 tissue-specific expression gene sets, and reporting a single, most over-represented TF found by each method. No information is given on why these particular sets were chosen, and no parameters (such as pseudocount, or background set) used for these other methods were reported. The test procedures used to evaluate ASAP [26] were similar, using 117 synthetic datasets, and only one real ChIP-PET dataset.

## Conclusions

Our novel regression-based motif enrichment analysis approach shows great promise when the available biological signal is believed to be direct evidence of DNA-binding, such as ChIP data. The utility of our novel method in MEA applications utilizing indirect evidence of DNA-binding (e.g., mRNA expression data) is still to be determined. The Clover algorithm appears very effective, but it requires the user to define a set of genes. In cases where this is done by the user partitioning genes according to a biological signal, the PASTAA algorithm may be a better choice. Our results on partition maximisation suggest that PASTAA may fail on some datasets where its built-in partitioning constraints are inappropriate. A reasonable approach would be to use all three algorithms (LR, Clover and PASTAA) and look for concordance among their predictions. All algorithms developed, including our novel LR algorithm are available on-line at http://bioinformatics.org.au/ame/.

## Authors' contributions

RCM developed the software, performed the experiments and drafted the manuscript. TLB developed the mathematical framework, assisted in the design of the study and revised the manuscript. All authors read and approved the final manuscript.
